# A Side-by-Side Comparison of Wildtype and Variant Melanocortin 1 Receptor Signaling with Emphasis on Protection against Oxidative Damage to DNA

**DOI:** 10.3390/ijms241814381

**Published:** 2023-09-21

**Authors:** Sonia Cerdido, José Sánchez-Beltrán, Ana Lambertos, Marta Abrisqueta, Lidia Padilla, Cecilia Herraiz, Conchi Olivares, Celia Jiménez-Cervantes, José C. García-Borrón

**Affiliations:** 1 Department of Biochemistry, Molecular Biology and Immunology, School of Medicine, University of Murcia, 30120 Murcia, Spain; sonia.cerdidoo@um.es (S.C.); jose.sanchezb@um.es (J.S.-B.); ana.lambertos@um.es (A.L.); marta.ag@um.es (M.A.); lidia.padillap@um.es (L.P.); ceciliahs@um.es (C.H.); mcolisan@um.es (C.O.); celiajim@um.es (C.J.-C.); 2 Instituto Murciano de Investigación Biosanitaria IMIB-LAIB, El Palmar, 30120 Murcia, Spain

**Keywords:** melanocortin 1 receptor (MC1R), variant MC1R, melanoma, signaling, cAMP, extracellular signal-regulated kinases 1 and 2 (ERK1/2), proliferation, DNA damage

## Abstract

Common variants of the *MC1R* gene coding the α-melanocyte stimulating hormone receptor are associated with light skin, poor tanning, blond or red hair, and increased melanoma risk, due to pigment-dependent and -independent effects. This complex phenotype is usually attributed to impaired activation of cAMP signaling. However, several MC1R variants show significant residual coupling to cAMP and efficiently activate mitogenic extracellular signal-regulated kinase 1 and 2 (ERK1/2) signaling. Yet, residual signaling and the key actions of wildtype and variant MC1R have never been assessed under strictly comparable conditions in melanocytic cells of identical genetic background. We devised a strategy based on CRISPR-Cas9 knockout of endogenous *MC1R* in a human melanoma cell line wildtype for *BRAF*, *NRAS* and *NF1*, followed by reconstitution with epitope-labeled *MC1R* constructs, and functional analysis of clones expressing comparable levels of wildtype, R151C or D294H MC1R. The proliferation rate, shape, adhesion, motility and sensitivity to oxidative DNA damage were compared. The R151C and D294H RHC variants displayed impaired cAMP signaling, intracellular stability similar to the wildtype, triggered ERK1/2 activation as effectively as the wildtype, and afforded partial protection against oxidative DNA damage, although less efficiently than the wildtype. Therefore, common melanoma-associated MC1R variants display biased signaling and significant genoprotective activity.

## 1. Introduction

The *MC1R* (MIM# 155555, Ensembl ID ENSG00000258839) encoding for the melanocortin 1 receptor is responsible for much of the variation in skin and hair pigmentation in the normal population [1]. It is a highly polymorphic gene, with over 200 coding region variants described to date [2]. Several MC1R variants, such as R151C and D294H, are common in individuals of Caucasian descent and are associated with the so-called RHC phenotype characterized by light skin, blond or red hair, poor tanning ability, propensity to sunburn, and increased melanoma risk [1,2,3,4]. Extensive genetic epidemiology analyses estimated a prevalence around 60% for carriers of any *MC1R* variant [5]. Thus, although its penetrance is low-to-moderate, the contribution of *MC1R* variants to melanoma burden is important, as it confers a 60% higher risk to carriers [5].

MC1R is a G protein-coupled receptor (GPCR) expressed in melanocytes and melanoma cells, whose activity is positively regulated by the peptide agonists α-melanocyte-stimulating hormone (αMSH) and adrenocorticotropin, derived from the precursor polyprotein proopiomelanocortin. MC1R is a unique GPCR in that it is also negatively regulated by endogenous peptide ligands, the Agouti signaling protein and β-defensin [6,7,8]. Signaling from the MC1R is complex. MC1R is a Gs-coupled GPCR and most, if not all, its physiological actions are most often thought to be mediated by activation of the cAMP pathway, either through cAMP-dependent PKA activation or through the engagement of EPACs [9,10] Of note, MC1R shows significant constitutive activity and stimulates cAMP synthesis in the absence of agonists [11].

Skin cells are exposed to mutagenic ultraviolet solar radiation (UVR) [12,13,14,15]. A causal link between UVR-induced DNA damage and skin carcinogenesis, particularly melanomagenesis, has been firmly established, as shown by a significant association between melanoma and intense occasional exposure to UVR leading to sunburns during childhood. MC1R signaling is critically involved in adaptive cutaneous responses to UVR [2]. The current consensus posits that the protective effect of MC1R is due to a combination of pigmentation-dependent and -independent factors. The pigmentation-dependent component is accounted for by a switch from basal production of reddish pheomelanins to the synthesis of darker brown-black eumelanins. Whereas eumelanin is a photoprotective pigment due to its absorption properties in the UVR spectrum and its free-radical-scavenging properties, pheomelanin is a photosensitizer promoting production of reactive oxygen species (ROS) not only upon exposure to UVR, but also in the dark [16,17,18]. Accordingly, pheomelanins promote melanomagenesis in mice with a conditional *BRAF*-mutant allele [19]. Importantly, the RHC *MC1R* alleles analyzed to date have been shown to correspond to partial loss-of-function proteins with various degrees of residual functional coupling to the cAMP cascade (reviewed in [2]). Because the eu-pheomelanin switch depends on activation of the cAMP pathway, it is carried out efficiently only by wildtype (WT) MC1R, and it is impaired in carriers of MC1R variants. Accordingly, the association of variant *MC1R* alleles and increased melanoma risk relies, at least partially, on this pigmentation-dependent effect. However, several studies show that pigmentation-independent actions also contribute to the overall protection afforded by MC1R [5]. Indeed, stimulation of WT MC1R activates antioxidant defenses to reduce oxidative stress in melanocytes and leads to induction of the DNA repair pathways [20,21]. These actions are also thought to rely on cAMP signaling by MC1R. Thus, WT MC1R may promote genomic stability by (i) activating eumelanogenesis to shield nuclei from UVR, (ii) lowering oxidative stress, and (iii) triggering a DNA damage response, which is consistent with the lower mutation load in *MC1R*-WT compared with *MC1R*-variant melanomas [15].

However, work from several laboratories has shown that MC1R signaling is pleiotropic, as it involves not only activation of the cAMP pathway, but also of the peroxisome proliferator activated receptor (PPARγ) pathway [22,23], the PI3K pathway [22,24,25], and the extracellular signal regulated protein kinases ERK1 and ERK2 [26]. ERK1/2 signaling is normally initiated by peptide growth factors binding to receptor tyrosine kinases. Within melanocytes, this triggers an intracellular signaling module consisting of the sequential activation of NRAS GTPase and the kinases BRAF, MEK and ERK1/2. The relevance of this pathway is underlined by the fact that hyperactivity of the ERKs through mutations in NRAS, its negative regulator NF1, or its downstream effector BRAF are causally related to ~75% of sporadic melanomas [27]. Most notably, common MC1R variants with an impaired but not absent ability to trigger the cAMP pathway efficiently activated ERKs when expressed in heterologous cells or in melanoma cells [26]. Overall, these cAMP-independent pathways downstream of MC1R have the potential to contribute to the regulation of key aspects of melanocyte biology relevant for the malignant transformation of melanocytes and for the acquisition of a metastatic phenotype. For instance, the effects of MC1R on cellular proliferation [28,29,30,31,32], invasion [29], and protection against DNA oxidative damage [25] have been reported.

Despite the potential physiological relevance of non-canonical MC1R signaling and of non-pigmentary actions of MC1R, the signaling potential of WT and variant MC1R have never been assessed under strictly comparable conditions in melanocytic cells of identical genetic background expressing similar levels of the receptor protein. Our aim was to perform this comparative study so as to clarify unambiguously the residual signaling of frequent melanoma-associated MC1R variants, as well as the effects of WT or variant MC1R expression and activation on cell proliferation, shape, and motility, and on protection against oxidative DNA damage.

## 2. Results

### 2.1. Generation of MC1R Knockout Cells (MC1R-KO) and Reconstitution with Defined MC1R Variants

**Figure 1:**
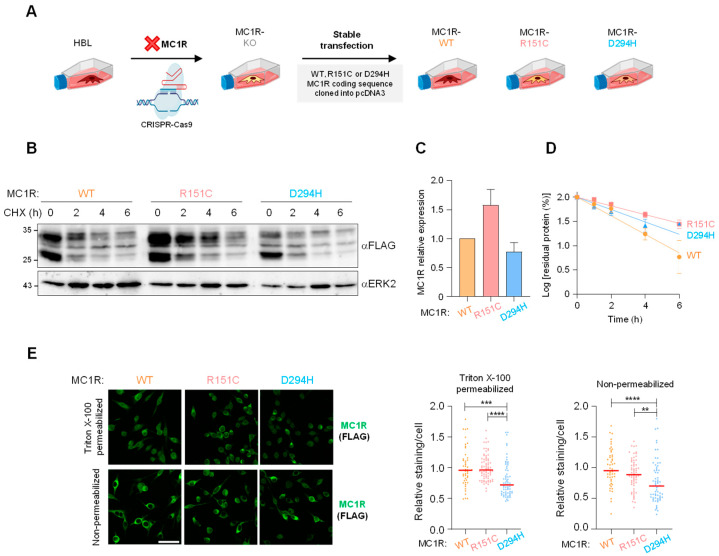
Stable expression of WT and variant MC1R in melanoma cells of identical genetic background. (**A**) Strategy for the generation of HBL human melanoma cell-derived clones expressing a single and defined variant of the MC1R. See text and Supplementary Figure S1 for details. (**B**) Expression and intracellular stability of the WT, R151C and D294H forms of MC1R. Cells were treated with 0.1 mM cycloheximide for the indicated times, detergent-solubilized, electrophoresed, and analyzed for MC1R with anti-flag. (**C**) Steady-state level of expression of WT and variant MC1R. Detergent-solubilized cell extracts were analyzed for MC1R expression via Western blot. The intensity of the MC1R band was corrected for protein loading using β-actin (ACTB) as the loading control. Results are normalized to the expression of WT MC1R and are the mean ± sem for 7 independent experiments. (**D**) Semi-logarithmic plots for the estimation of the rate of decay of WT or variant MC1R in live HBL cells. The intracellular half-lives of the different receptor forms, estimated from the slopes of the adjusted linear plots, are indicated (mean ± sem, n ≥ 3). (**E**) Confocal micrographs of HBL cells expressing defined MC1R variants. MC1R was immunostained with anti-FLAG, with or without a 15 min treatment with 0.4% Triton X-100 in PBS for permeabilization of the cell membrane. The graphs on the right show the MC1R staining intensity normalized to the cells expressing the WT receptor. **, *p* < 0.01; ***, *p* < 0.001; ****, *p* < 0.0001. Scale bar 50 µm.

In order to obtain melanocytic cells of identical genetic background but expressing defined variants of epitope-tagged MC1R, we first generated a MC1R-KO melanoma cell line using HBL melanoma cells (strategy depicted in Figure 1A). HBL cells belong to the triple-WT molecular subtype of melanomas (*NRAS*, *BRAF* and *NF1* WT) and they respond to stimulation with melanocortin agonists with strong increases in both cAMP and ERK1 and ERK2 activity [26]. First, expression of endogenous MC1R in these cells was abolished by CRISPR-Cas9 (Supplementary Figure S1), and clones were selected based on lack of a detectable cAMP response following stimulation with the synthetic agonist [Nle4, D-Phe7]αMSH (NDP-MSH, 100 nM, 30 min). Next, cells were stably transfected with cDNA encoding for one of three defined variants of MC1R, namely the WT and the R151C and D294H RHC-type variants, cloned into the pcDNA3 vector. In all cases, the flag epitope was fused in frame to the N terminus of the MC1R to yield proteins comparably reacting with a high-affinity antibody. Previous work has shown that the function of flag-tagged and native MC1R are similar if not identical [33]. Transfected cells were selected in geneticin-containing medium, and individual clones were analyzed for MC1R expression.

Clones expressing WT, R151C or D294H MC1R showed a consistent electrophoretic pattern in Western blots probed with an anti-flag monoclonal antibody (Figure 1B), with similar steady-state levels of WT and D294H, and a slightly higher expression of R151C (Figure 1C). WT and the RHC variants R151C and D294H were processed similarly, as shown by the presence of two forms of electrophoretic mobility corresponding to 25 (de novo protein) and 35 (glycosylated, mature form) kDa [34]. We also analyzed the intracellular stability of the three MC1R forms by blocking protein synthesis with cycloheximide 10^−4^ M final working concentration, as described by others [35], and by following the rate of disappearance of the MC1R signal in Western blots of extracts from cells lysed at different time points (Figure 1B). Semilogarithmic plots of the residual MC1R abundance as a function of the chase time allowed for the calculation of the intracellular half-life of the MC1R forms (Figure 1D). The R151C and D294H variants appeared slightly more stable than WT, as shown by half-lives of 2.1 ± 0.8 h for WT, 3.6 ± 0.7 h for R151C, and 2.6 ± 1.0 h for D294H, consistent with the somewhat higher steady state concentration of R151C (Figure 1B,D). Finally, to distinguish the native mature receptor in the plasma membrane of cells, and the intracellularly retained protein, likely due to impaired trafficking through the secretory pathway [36], we analyzed confocal micrographs immunostained for MC1R in the presence or absence of the permeabilizing agent Triton X-100 (Figure 1E). We confirmed similar expression levels for the receptor forms, as well as intracellular retention of R151C, as previously described [33]. Overall, these data demonstrated that the WT, R151C, and D294H forms of the MC1R were adequately and comparably expressed and processed in HBL human melanoma cells. Moreover, the slightly higher intracellular stability of the variant proteins compared with WT strongly suggested that R151C and D294H escaped degradation via the quality control machinery of the biosynthetic–secretory pathway. Thus, the cells expressing defined MC1R variants appeared suitable for functional analysis.

### 2.2. Signaling Downstream of WT and Variant MC1R

**Figure 2:**
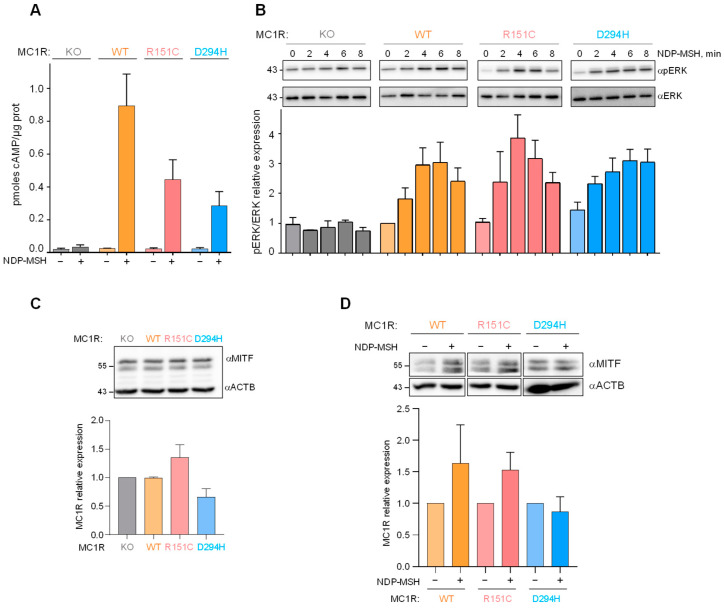
Functional coupling of WT and variant MC1R. Cells expressing the indicated MC1R variants were stimulated with 100 nM NDP-MSH for the times shown and analyzed for (**A**) intracellular cAMP and (**B**) ERK activation. The upper Western blots are representative of three independent experiments, and the lower bar graphs represent the quantification of the active ERK intensity, normalized to the non-stimulated control (0 min timepoint, mean ± sem, n = 3). (**C**) Steady-state levels of MITF in unstimulated cells expressing the indicated MC1R forms. The image is representative of five independent Western blots, whose quantification is shown below as a bar graph (values normalized to the MC1R-KO expression, results given as mean ± sem, n = 5). (**D**) Changes in MITF expression upon stimulation of cells expressing the indicated MC1R form with 100 nM NDP-MSH for 48 h. Representative blots are shown at the top, and the fold change in band intensity, normalized to each non-stimulated control, is shown below (mean ± sem, n = 3).

Next, we compared the ability of the MC1R forms to trigger the cAMP and ERK pathways upon stimulation with 100 nM NDP-MSH, the lowest agonist concentration achieving a maximal stimulation of MC1R in HBL cells, according to our previous studies [26]. After a 30 min challenge with the agonist, the cAMP contents were roughly 50% for R151C and 25% for D294H, compared with cells expressing the WT receptor (Figure 2A). Therefore, both R151C and D294H significantly stimulated cAMP synthesis, although with lower potency than WT. Conversely, WT, R151C and D294H achieved a comparable stimulation of the ERKs, both in terms of maximal activation and of the kinetics of the process (Figure 2B), except maybe for a slightly more sustained activation downstream of the D294H variant. As expected, NDP-MSH did not activate the cAMP or ERK pathways in MC1R-KO cells. In summary, these data obtained in melanocytic cells of identical genetic background confirmed previous studies performed in heterologous expression systems (reviewed in [2]), and showed that common melanoma-associated RHC alleles are not canonical loss-of-function proteins, but biased signaling receptors preferentially activating the mitogenic ERK pathway.

Microphthalmia-associated transcription factor (MITF) is a master regulator of key aspects of the biology of melanocytes and melanoma cells, including survival, differentiation, and proliferation (reviewed in [37,38]). MITF expression is activated by cAMP via PKA-dependent phosphorylation of CREB. Moreover, phosphorylation by ERKs targets MITF for proteolytic degradation. Since MITF expression and intracellular stability are regulated by the main signaling pathways triggered by MC1R, we analyzed the effects of the different MC1R forms on MITF levels. As shown in Figure 2C, basal MITF levels were comparable in unstimulated cells expressing WT, R151C or D294H. However, when cells were stimulated with 100 nM NDP-MSH for 48 h, MITF expression was augmented in cells expressing WT MC1R and to a lesser extent R151C, but not in cells expressing D294H (Figure 2D). This may reflect the signaling bias of the different MC1R forms, in that upon stimulation with an MC1R agonist, D294H showed the lower MITF expression-promoting cAMP signaling on one hand, and the more sustained MITF degradation-promoting ERK activity on the other.

### 2.3. Effects of MC1R Genotype on Melanoma Cell Proliferation

**Figure 3:**
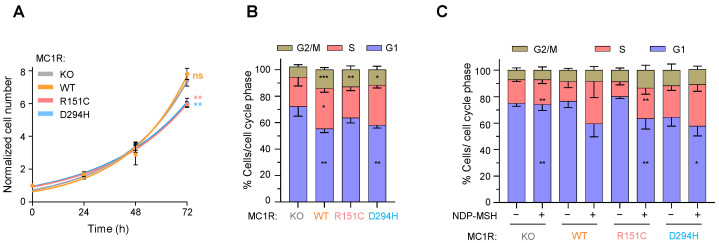
MC1R activation modulates proliferation and cell cycle progression. (**A**) Growth curves of MC1R clones cultured in 2D and complete medium (DMEM + 10% FCS) determined by manual cell counting. Equal numbers of cells were seeded, cells were allowed to attach for 24 h (time 0) and counted every 24 h. Growth was represented as fold increase in cell number relative to the initial number in the 0-time point. The statistical significance of the cell numbers in MC1R-expressing cells compared with MC1R-KO cells at 72 h is shown. (**B**) FACS analysis of MC1R clones cultured in total medium (DMEM + 10% FCS) for three days. Results are given as mean ± sd (n = 5). Stars indicate the statistical significance of each phase compared with the same phase in MC1R-KO cells. (**C**) FACS analysis of MC1R clones cultured under FCS-starved conditions (DMEM and no FCS) for two days in the presence or absence of 100 nM NDP-MSH. For each receptor form, the stars within the bars of the NDP-MSH condition indicate the statistical significance of each phase compared with the same phase in the corresponding untreated control (results given as mean ± sd, n ≥ 3; ns, not significant). *, *p* < 0.05; **, *p* < 0.01; ***, *p* < 0.001.

We analyzed the proliferation of cells expressing the various MC1R forms in three different culture conditions: (i) complete growth medium supplemented with 10% FCS, (ii) FCS-deprived media containing only 1% added serum, and (iii) FCS-deprived media supplemented with 100 nM NDP-MSH. Cell numbers were determined by manually counting viable cells in the presence of trypan blue, and the distribution of the cell populations in the different phases of the cell cycle was estimated by FACS after DNA staining with propidium iodide. As shown in Figure 3A and Table 1, the proliferation curves for cells cultured in complete medium supplemented with 10% FCS were similar in all cases, except for a somewhat slower rate for cells expressing variant MC1R. Thus, when the culture medium was enriched in mitogens provided by FCS, the presence or absence of MC1R, or the MC1R variant expressed by the cells, had only a minor effect on the doubling time of the cultures (Figure 3A and Table 1). On the other hand, the cell cycle profiles of WT, R151C and D294H cells grown in the presence of 10% FCS displayed subtle differences compared with MC1R-KO cells (Figure 3B), in that the percentage in the S + G2 phases of the cycle was higher for cells expressing any receptor form, whereas for MC1R-KO cells, the fraction of cells in G1 was higher despite a doubling time similar to the WT and slightly but significantly shorter than R151C or D294H (Table 1).

On the other hand, when the FCS concentration was lowered to 1%, the doubling time of all the cells increased roughly two- to three-fold, relative to cells grown in 10% FCS, consistent with a limited availability of mitogenic factors (Figure 3C). For cells expressing WT or R151C, FACS analysis revealed a decreased population of cells in the S phase of the cell cycle in the low FCS condition compared with the 10% FCS medium, consistent with their increased doubling times (Table 1). Of note, addition of NDP-MSH to cultures growing in low serum medium augmented the doubling time of cells expressing WT more than 2-fold, but not variant MC1R (Table 1). Moreover, NDP-MSH augmented the percentage of WT or R151C cells in the S phase of the cell cycle, whereas, as expected, the cell cycle profile of MC1R-KO cells was largely insensitive to the presence or absence of NDP-MSH. These observations are consistent with a previous report [32] showing that cAMP signaling downstream of MC1R impairs melanoma cell growth by inhibiting the activity of cdc25B, a phosphatase that removes the inhibitory phosphorylation of cyclin-dependent kinase 1 (CDK1) to promote the cell cycle’s progression to mitosis. In light of this report, it is conceivable that strong cAMP signaling downstream of WT, but not variant MC1R, may inhibit cdc25B activity to interfere with CDK1 activation and the completion of mitosis. In any case, the effects of the *MC1R* genotype on cell growth under different conditions of serum and/or melanocortin concentration appear complex, and deserve further study.

### 2.4. Effects of MC1R Genotype on Melanoma Cell Shape and Motility

**Figure 4:**
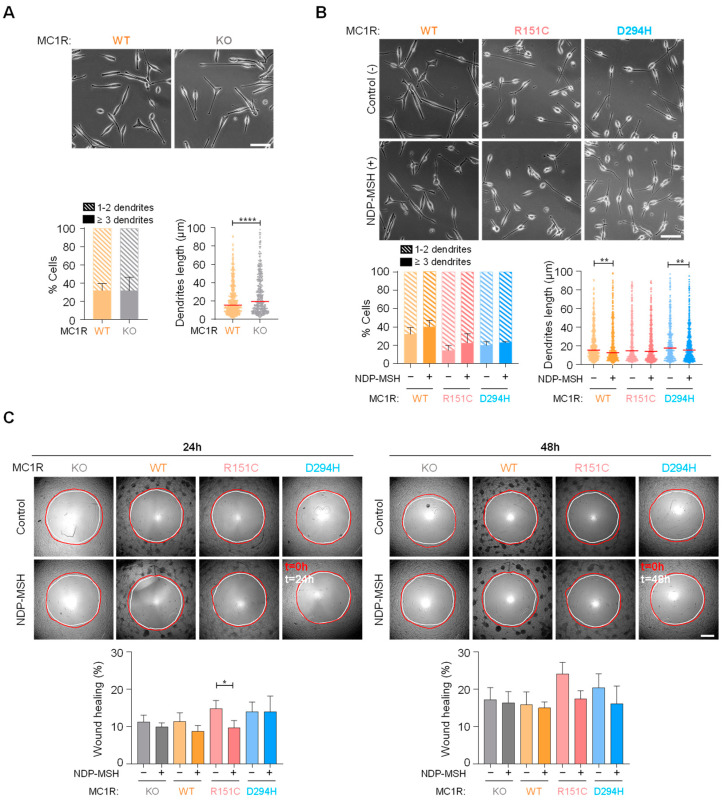
Effect of the *MC1R* genotype on cell shape and motility. (**A**) Effect of WT MC1R on shape and dendricity. MC1R-KO cells or cells expressing WT MC1R were grown in complete medium with 10% FCS. Micrographs were taken using an Eclipse TS2 microscope with 20x objective lenses, scale bar 50 µm. A quantitative analysis of the number and length of dendrites per cell in randomly selected images is shown below the micrographs (at least 100 cells per condition were analyzed, and the results are given as median ± SEM for length or mean ± SEM for number, n = 3). (**B**) Effect of NDP-MSH on shape of cells expressing WT or variant MC1R. When required, cells were treated for 48 h with 100 nM NDP-MSH before acquisition and analysis of the micrographs (100 cells quantified per condition; the scale bar and results are formatted as in (**A**)). (**C**) Basal and MC1R-agonist induced migration of cells expressing the different MC1R forms. Cells were seeded on Oris™ 96-well plates with silicon stoppers in serum-reduced medium, and if required were treated with 100 nM NDP-MSH for 24 h. The stoppers were removed, and images were taken at 24 h or 48 h after removal of the stoppers. Representative images are shown, along with the quantification of wound healing at 24 h or 48 h (results are given as mean ± SEM, n = 3). *, *p* < 0.05; **, *p* < 0.01; ****, *p* < 0.0001.

We compared the morphology of HBL cells expressing (or not) MC1R in terms of the number and length of dendrites. To this end, cells were grown on 6-well plastic plates in 10% FCS-containing medium, phase-contrast micrographs were randomly collected, and the images were analyzed with ImageJ (Figure 4A). Cultures consisted mostly of bipolar cells, with roughly 30% of cells presenting three or more dendrites, and no significant difference in this percentage between WT and MC1R-KO cells was observed. Conversely, MC1R-KO cells exhibited significantly longer dendritic processes. Concerning the effects of the MC1R status, cells expressing the R151C or D294H variant tended to display fewer dendrites than cells expressing WT MC1R, although this trend did not reach significance. NDP-MSH treatment had little if any effect on the number of dendrites, but tended to decrease their length in cells expressing any MC1R form, and this trend reached significance for cells expressing WT or D294H (Figure 4B).

Next, the motility of cells expressing the various MC1R variants was compared by means of 2D circular wound assays (Figure 4C). In the absence of an agonist, the rate of wound closure was the same for MC1R-KO cells and cells expressing any MC1R form. NDP-MSH tended to decrease cell motility, particularly at the 24 h time point, but the effect was modest, and reached statistical significance only for R151C. In summary, the presence or absence of MC1R had little effect on the shape and motility of HBL human melanoma cells.

### 2.5. Protection of DNA from Oxidative Stress

Although the ability of WT MC1R to induce repair of various DNA lesions is well established [39,40], it remains unclear whether the frequent *MC1R* alleles associated with increased skin cancer risk encode for proteins that have lost completely the potential to activate DNA repair pathways or, alternatively, maintain residual levels of this potential. Oxidative stress is toxic to DNA as it leads to base oxidation, formation of abasic sites, and single- (SSB) or double-strand breaks (DSB) (reviewed in [41,42]). It has been reported that, in melanocytes of WT but not variant *MC1R* genotypes, αMSH protects against oxidative DNA lesions and reduces the phosphorylation of histone H2AX, a marker directly correlated with DSBs, at least partially, via cAMP-dependent induction of the base excision repair pathway [20]. However, our previous work showed a significant induction of oxidatively-induced DNA strand break repair downstream of variant MC1R in *BRAF*-mutated human melanoma cells [25]. These different results can most likely be accounted for by the different cellular contexts. Thus, it was of interest to compare the protection against oxidative lesions afforded by WT versus variant MC1R in identical cellular backgrounds.

**Figure 5:**
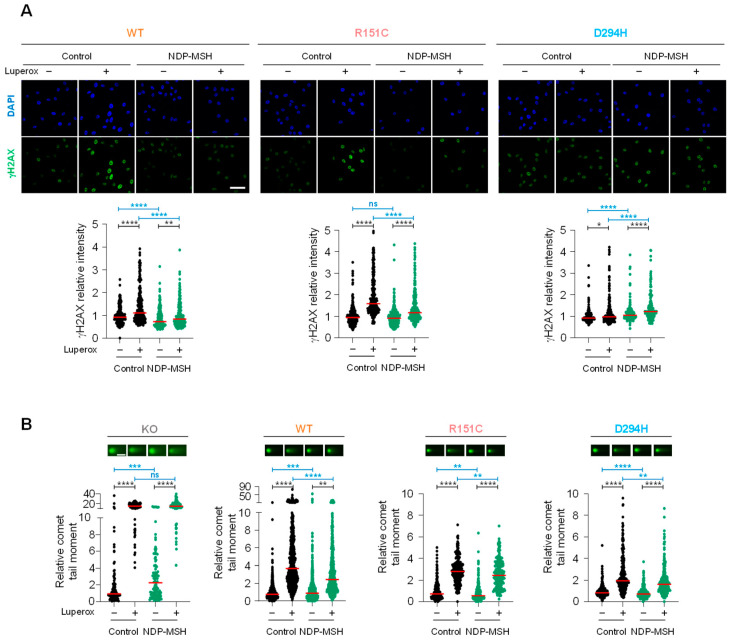
Genoprotective action against oxidative stress of the different MC1R forms. (**A**) γH2AX immunostaining of control cells and cells challenged with Luperox (150 µM, 30 min) with or without a previous treatment with NDP-MSH (100 nM, 48 h). The confocal images correspond to one of two independent experiments with comparable results. Scale bar 50 µm. For each experiment, at least 100 cells were randomly selected and analyzed for staining intensity. The plots below show the median of the staining intensity of cells, normalized to the control condition (no treatment with NDP-MSH or Luperox). (**B**) Neutral comet assay. In this case, cells pretreated or not with NDP-MSH were challenged with 100 µM Luperox for 20 min. Two independent experiments were performed with consistent results. *, *p* < 0.05; **, *p* < 0.01; ***, *p* < 0.001; ****, *p* < 0.0001. Scale bar 25 µm.

Since DSBs can be labeled by phosphorylated histone H2AX (γH2AX) immunostaining or specifically visualized by comet assays performed under neutral conditions, we treated our cell lines with the semi-stable peroxide Luperox at a concentration able to increase intracellular ROS and therefore to induce DNA damage without significantly impairing cellular viability [25]. We analyzed the genotoxic effect via detection of γH2AX foci or neutral comet assays (Figure 5).

For cells expressing either WT or R151C MC1R, short Luperox pulses augmented the burden of DSBs as detected by γH2AX staining (Figure 5A) or neutral comet assays (Figure 5B). Previous stimulation of MC1R with NDP-MSH partially prevented this increase in DSB abundance. Intriguingly, cells expressing the D294H form seemed relatively insensitive to Luperox when they were analyzed via γH2AX staining (Figure 5A, right panel), although their behavior was consistent with the one of cells expressing WT or R151C when they were analyzed for DSBs by comet assay (Figure 5B). It is also worth noting that of the various cell lines analyzed by comet assays, MC1R-KO cells were the most sensitive to the Luperox challenge in the absence of pretreatment with NDP-MSH, and that NDP-MSH was not capable of protecting these cells against oxidative DNA fragmentation (Figure 5B). This indicated that at least in our cell culture conditions, expression of MC1R, either WT or variant, conferred significant protection against oxidative insults, even in the absence of exogenous agonists. In summary, these data showed that at least two of the most frequent MC1R variants, R151C and D294H, displayed significant residual ability to protect against oxidative DNA damage.

## 3. Discussion

The *MC1R* genotype is a major genetic determinant of melanoma and nonmelanoma skin cancer risk, with frequent variants such as R151C or D294H increasing the odds of developing these diseases [1,2,3,4,5]. Analysis of the observed versus expected *MC1R* transcript variation across the gnomAD Database (Genome Aggregation Database) shows that the observed missense MC1R variants are more abundant than the expected probability predicted for human genome. Of note, MC1R variants p.Arg151Cys and p.Asp294His were among the top 10 missense variants across 383 different SNPs found (from near 300.000 exomes and genomes analyzed), with an allele frequency of 4.48 × 10^−2^ and 9.16 × 10^−3^, respectively. R151 and D294 are highly conserved residues across mammals and other organisms, and their variations (rs1805007 and rs1805009) have been observed in multiple ethnic backgrounds with the highest frequencies in individuals of European ancestry. These variants are present in ClinVar (IDs 14,312 and 14307) and have been associated with increased risk of melanoma by several studies. Indeed, a meta-analysis of MC1R carriers for these variants reported odds ratio values consistent with their high-risk association with developing melanoma [5,43]. Accordingly, to identify the molecular bases of this association is a goal of the utmost importance, particularly in light of the high and increasing incidence of skin cancers [44]. This aim is heavily dependent on a deep understanding of the functional differences between WT and variant MC1R, but an accurate study of these differences is complicated by the likely dependence of key biological actions of MC1R on the cellular context. For instance, dominant negative effects in *MC1R* heterozygotic cells have been reported [33,45]. Moreover, modulation of MC1R downstream signaling is likely to depend on the mutational landscape of melanoma cells [46], and melanocyte-specific responses such as regulation of MITF levels cannot be properly analyzed in heterologous cells engineered to express specific MC1R forms. To circumvent these limitations, we used a strategy to express single and defined MC1R variants in an identical melanocytic background, and we performed an accurate comparison of the signaling potential of WT and variant MC1R in melanocytic cells of identical genetic background. Our approach involved the permanent and complete knockout of endogenous MC1R in HBL cells, a human melanoma cell line of the triple WT molecular subtype, followed by stable transfection with WT, R151C or D294H constructs. The low basal ERK activity provided by the *NRAS*, *BRAF* and *NF1* WT genotype and the low cAMP levels in unstimulated, resting cells allowed for an accurate analysis of activation of the ERK and cAMP pathways.

Our results demonstrate that WT MC1R is positively coupled to the cAMP and ERK signaling pathways. Moreover, our data firmly establish that the common variants R151C and D294H are not classical loss-of-function forms, but rather are biased signaling variants with different degrees of residual signaling to the differentiation-promoting cAMP pathway on one hand and retention of full signaling to the mitogenic ERK module on the other hand. Likely because of this signaling bias, the behavior of melanoma cells of identical background but harboring different MC1R forms showed subtle but significant differences in relevant biological properties such as proliferation, cell cycle progression, and response to the genotoxic action of oxidative stress. The functional coupling of the variants to other signaling pathways reported to mediate certain MC1R actions, such as the PPARγ pathway [22,23] or the PI3K pathway [22,24,25], has not been investigated, and will be further analyzed in subsequent studies.

Not surprisingly, the results presented above suggest that the different variant alleles are not functionally equivalent. Conceivably, the cAMP-dependent actions of MC1R would be more significantly impaired in carriers of RHC variants with lower residual coupling to the cAMP cascade. On the other hand, for variants such as R151C and D294H maintaining full ERK activation potential, the ERK-dependent effects of MC1R should be mostly preserved, with little if any changes compared with WT. This might have a substantial effect on the levels of MITF in melanocytes and melanoma cells, as cAMP signaling increases *MITF* gene expression, whereas ERK activity promotes MITF protein degradation [37,38]. Interestingly, in keeping with this hypothesis, we found a differential response of MITF levels to MC1R activation in cells expressing either WT, R151C or D294H. In any case, both R151C and D294H are most likely functionally different from variant alleles, leading to complete loss of signaling potential, particularly those resulting in aberrant trafficking with complete retention in internal cellular compartments, or the inability to bind agonists at physiological concentrations such as the single amino acid mutants S41F or R162P (reviewed in [2], or presenting premature stop codons leading to early truncation of the protein [47].

Given that UVR is the main external etiologic factor in melanoma, the signaling and functional alterations of the RHC alleles might be particularly important in the context of protection against the genotoxic action of solar radiation. The UV components of solar radiation trigger various types of DNA damage, accounting for up to 1 × 10^5^ DNA lesions/cell/day [48]. These lesions are cleared by a series of functionally interwoven pathways that share specific components, and whose regulation is only partially understood. Energetic UVB is mostly associated with formation of bulky cyclobutane pyrimidine dimers and pyrimidine (6–4) pyrimidinone photoproducts targeted by the nucleotide excision repair (NER) pathway. Following early reports of MC1R-dependent clearance of UVB-induced DNA photoproducts [22], the role of MC1R-mediated, cAMP-dependent induction of NER has been confirmed (reviewed in [49]). Accordingly, deficient cAMP signaling downstream of variant MC1R should impair NER-mediated clearance of DNA lesions caused by UVB, a notion fully supported by extensive analyses of the mutational burden in melanomas of WT or variant MC1R genetic background [15]. On the other hand, the less energetic UVA targets DNA indirectly, via generation of ROS that oxidize bases such as guanine and cause SSBs and DSBs (reviewed in [50]). Oxidative DNA lesions such as guanine oxidation and SSBs are cleared by the base excision repair pathway, and extremely toxic DSBs are repaired either by homologous recombination or by the error-prone non-homologous end joining pathway. Protection against oxidative lesions by MC1R agonists has been reported for WT MC1R, where it was most likely dependent on cAMP signaling [20]; however, a recent report showed its occurrence in melanoma cells of variant *MC1R* genotype, where it relied most likely on cAMP-independent signaling [25]. Here, we unambiguously showed that activation of either WT or R151C significantly reduced the DSB burden caused by oxidative stress in melanoma cells of the triple WT molecular subtype and a comparable genetic background. The situation was less clear-cut for D294H, since a modest although significant protection was detected in neutral comet assays, but not in γH2AX immunostained cells. The reason for this discrepancy is unclear, and needs further analysis. It also remains to be investigated whether the residual activation of the cAMP pathway downstream of R151C or D294H is responsible for the protective effect of these variants in the HBL cellular background, or, alternatively, is mediated by other signaling pathways such as the ERK cascade or the PI3K-AKT module. The interesting possibility that the choice of the pathway leading to genoprotection against oxidative lesions downstream of MC1R might depend on the cellular context, for instance, on the presence or absence of constitutive hyperactivation of the ERK pathway, should also be considered. In any case, the significant genoprotection against oxidative insults afforded by variant MC1R has now been formally demonstrated by the results reported above. This may have important biological consequences, as it should help MC1R variant melanocytes and melanoma cells to cope with the oxidative stress resulting from the pheomelanic phenotype associated with their *MC1R* genotype.

## 4. Materials and Methods

### 4.1. Reagents

Protease or phosphatase inhibitors and common laboratory reagents were from Sigma (St. Louis, MO, USA), Calbiochem (Darmstadt, Germany), Merck (Darmstadt, Germany) or Prolabo (Barcelona, Spain), unless specified otherwise. Lipofectamine 2000 was from Invitrogen. Opti-MEM I and other cell culture reagents were from Gibco (Gaithersburg, MD, USA). Reagents for SDS-PAGE and Western blot were from Bio-Rad (Richmond, CA, USA).

### 4.2. Generation of CRISPR/Cas9-Based MC1R-KO Cells and Reconstitution with Defined MC1R Variants

Since the crRNA and tracrRNA can be fused together to create a chimeric, single-guide RNA (sgRNA) [51], we designed several sgRNAs, consisting of 20-nt guide sequence base pairs which must immediately precede a 5′-NGG PAM (Supplementary Figure S1 and Table S1). Efficiencies and potential off-targets were determined using the Breaking-Cas web tool (http://bioinfogp.cnb.csic.es/tools/breakincas (accessed on 15 October 2018)) [52]. According to these values, we selected sgRNA4 for the experiments. To generate the sgRNA4 expression construct, sgRNA4 oligos (Dharmacon; Lafayette, CO, USA) were cloned into the pSpCas9(BB)-2A-Puro Cas9 Nuclease Expression plasmid with Puromycin mammalian antibiotic selection marker, v2.0 (Addgene plasmid ID: 62988), for co-expression with Cas9, by annealing the top and bottom oligos and ligation into the plasmid. Next, HBL human melanoma cells (a cell line obtained at the Laboratory of Oncology and Experimental Surgery of the Free University of Brussels, a kind gift from Prof G. Ghanem) were transfected with 1.0 µg of the CRISPR plasmid (pSpCas9(sgRNA)) and 2.0 µg of Lipofectamine^TM^ 2000. For negative control cells, we transfected the original vector with no insert. After incubation at 37 °C for 72 h, puromycin-resistant clonal cells were selected with 1 µg/mL puromycin. Confirmation and selection of positive clones was performed by sequencing and a cAMP production assay upon NDP-MSH stimulation. Stable transfectants expressing the desired MC1R forms were cultured in the presence of 800 μg/mL G418 sulfate. We used flag-tagged WT or RHC-variant (R151C, D294H) MC1R-pcDNA3 constructs [33]. Protein expression was ascertained by Western blotting with the anti-FLAG M2 monoclonal antibody from Merck.

### 4.3. Cell Culture and Analysis of Proliferation and Cell Cycle Progression

Cells were cultured in DMEM-GlutaMax supplemented with 10% FCS (unless otherwise specified), 100 U/mL penicillin and 100 µg/mL streptomycin, in the continuous presence of geneticin, in a water-saturated atmosphere containing 7.5% CO_2_. To study cellular proliferation, equal numbers of cells (3 × 10^4^) were seeded on 12-well plates in DMEM-GlutaMax supplemented with either 10% FCS, 1% FCS, or 1% FCS + 100 nM NDP-MSH. Cells were allowed to attach and recover for 24 h, then manually counted using a hemocytometer at different times from 24 to 96 h. Doubling times were calculated by nonlinear regression using an exponential growth equation and GraphPad Prism Software (https://www.graphpad.com, San Diego, CA, USA). For cell cycle analysis, cells fixed in ethanol 70% in PBS were pelleted, resuspended in PBS, and further treated with 100 µg/mL RNase A and 40 µg/mL propidium iodide (PI). At least 10^4^ cells were analyzed in a LSRFortessa X-20 cytometer (BD Biosciences, Franklin Lakes, NJ, USA), using ModFit LT^TM^ software (https://www.vsh.com/products/mflt/).

### 4.4. Analysis of Cell Morphology and Migration

Cells (usually 5 × 10^4^) were seeded on 6-well plates in complete medium with 10% FCS. When required, 24 h later cells were serum-deprived for 12 h and stimulated with 100 nM NDP-MSH. Images were taken after 48 h of treatment, using an Eclipse TS2 microscope with 20× objective lenses. Quantitative analysis of cell morphology was performed using Image J, by comparing the number and length of dendrites per cell. Cellular migration was studied using the Oris^TM^ Universal Migration Assembly Kit (CMAU505), following manufacturer’s instructions. Briefly, 6.5 × 10^4^ cells were seeded on Oris™ 96-well plates with silicon stoppers in serum-reduced medium, and if required were treated with 100 nM NDP-MSH for 24 h. The stoppers were removed, and images were taken at the beginning of migration (t = 0 h) and at different points (24 or 48 h) as described above. The percentage of wound closure was determined by measuring the wound area (Image J) and normalizing to t = 0 h.

### 4.5. Analysis of DNA Integrity and Detection of DSBs

DNA integrity was assessed by means of comet assays and γH2AX immunostaining. The neutral comet assay was performed according to manufacturer’s protocol (Trevigen, Gaithersburg, MD, USA). Electrophoresis was performed in 1.5 M sodium acetate, 500 mM Tris base, pH = 9, for 30 min at 4 °C. After electrophoresis, the gels were stained with SYBR Green I Nucleic Acid Gel Stain™. Images were taken using a Nikon Eclipse TS2 microscope with 10× objective lens. Tail moments of at least 100 comets were randomly selected and measured using CASPLAB software (http://casplab.com/). For γH2AX immunostaining, cells were fixed in 4% formaldehyde and permeabilized with 0.5% Triton X-100. After blocking with BSA, samples were incubated overnight with an anti-γH2AX monoclonal antibody recognizing phospho-S139, at a 1:250 dilution (Abcam, Cambridge, UK, catalog number ab2893), followed by an Alexa 488-conjugated secondary antibody (ThermoFisher, Waltham, MA, USA, catalog number A-11070, 1:300 dilution, 1 h at room temperature). DNA was stained using DAPI and images were taken with a SP8 Leica laser scanning confocal microscope and software (Leica Microsystems GmbH, Wetzlar, Germany), using HCXPL APO CS 40× or 63× objective lenses. γH2AX fluorescence signal was quantified by calculating the pixel intensity in single cell nuclei relative to the nucleus area. At least 100 randomly selected cells per condition were quantified using ImageJ (rsb.info.nih.gov/ij).

### 4.6. Immunoblotting and Immunofluorescence

Western blotting was performed as described [34] using antibodies directed against the FLAG epitope (M2 monoclonal antibody, Sigma-Aldrich, catalog number A8592, working dilution 1:5000), ERK2 and phosphoERK1/2 (Santa Cruz Biotechnology, catalog numbers sc-1647 and sc-16982, working dilution 1:7500 and 1:5000, respectively), MITF (Cell Signaling Technology, Danvers, MA, USA, catalog number 12590, working dilution 1:3000), or β-actin (Sigma-Aldrich, catalog number A2066, working dilution 1:10,000). For MC1R immunostaining and signal quantification, 3 × 10^4^ cells were seeded on glass coverslips and grown for 48 h. Confocal MC1R detection was performed as previously described [33]. Briefly, after 10 min fixation with 4% paraformaldehyde in PBS, cells were permeabilized using a 15 min treatment with 0.4% Triton X-100 in PBS (if needed). MC1R was labeled after overnight incubation with anti-flag rabbit antibody (1:400 in PBS containing 4% BSA) followed by a 1h incubation at room temperature with Alexa 488-conjugated secondary antibody (ThermoFisher, catalog number A-11070, 1:700). For non-permeabilized cells, all steps after fixation were performed over an ice bed. Samples were mounted with DAKO medium (Glostrup, Denmark), and confocal micrographs were obtained with the SP8 Leica microscope. Around 100 randomly selected cells per condition were quantified with ImageJ.

### 4.7. Functional Assays

For cAMP measurements, cells grown on 12-well plates were serum-deprived for at least 3 h and stimulated with 100 nM NDP-MSH. The medium was aspirated, and the cells washed with 800 µL ice-cold PBS, lysed with 200 µL/well 0.1N HCl preheated at 70 °C, and scrapped. The mix was freeze-dried, washed with 100 µL H_2_O and freeze-dried again. cAMP was measured with a commercial competitive enzyme immunoassay from R&D Systems (catalog number KGE002B). Parallel dishes were used for protein determination with bicinchoninic acid. To estimate ERK activation, the levels of phosphorylated ERK (pERK) were analyzed via Western blot. Cells were solubilized in 75 µL PBS supplemented with PMSF 100 ng/mL, 1% Igepal and 1% phosphatase inhibitor mix from Calbiochem. Samples were centrifuged and a volume of supernatant containing 30 µg protein was electrophoresed and blotted as described. Blots were probed with an anti-pERK1/2 rabbit polyclonal IgG (Santa Cruz Biotechnology, Santa Cruz, CA, USA) and stained with a chemiluminescent substrate. Comparable loading was ascertained by stripping and reprobing the membranes with anti-ERK2. Quantification of band intensity was performed with ImageJ software (available at rsb.info.nih.gov/ij).

### 4.8. Statistical Analysis

All analyses were carried out using GraphPad Prism. An unpaired two-tailed Student’s *t*-test and one-way ANOVA with Tukey’s post-test for multiple comparisons were performed, as required. Unless otherwise specified, results are expressed as mean ± SEM, and *p* values were calculated using two-sided tests. *p* values of less than 0.05 were considered significant.

## Figures and Tables

**Table 1:** Differential effect of MC1R variants on the growth of human melanoma cells.

Culture Conditions	Doubling Time (h) ^1^			
	MC1R-KO	WT	R151C	D294H
10% FCS	21.5 ± 0.7	19.8 ± 1.5	26.5 ± 1.4	26.7 ± 0.6
1% FCS	64.6 ± 1.6 *	45.3 ± 2.8 *	56.0 ± 3.8 *	42.7 ± 2.4 *
1% FCS + 100 nM NDP-MSH	55.8 ± 6.4 *	>100	59.1 ± 8.3	41.3 ± 3.0 *

^1^ HBL human melanoma cells were engineered to express no MC1R (MC1R-KO) or the indicated MC1R variants. Doubling times were calculated by nonlinear regression of proliferation curves obtained for cells grown in DMEM supplemented with (i) 10% FCS, (ii) 1% FCS or (iii) 1% FCS + 100 nM NDP-MSH, as indicated. Results are given as mean ± sem., n ≥ 3. The t-test was used to compare the doubling times of each cell type with the value obtained for the same cells growing in 10% FCS-enriched medium (*, *p* < 0.05).

## Data Availability

Not applicable.

## Supplementary Materials

The following supporting information can be downloaded at: https://www.mdpi.com/article/10.3390/ijms241814381/s1.

## Abbreviation

DSB	Double-strand break
ERK	Extracellular signal-regulated protein kinase
FCS	Fetal calf serum
GPCR	G protein-coupled receptor
MC1R	Melanocortin 1 receptor
αMSH	α-melanocyte-stimulating hormone
MITF	Microphthalmia-associated transcription factor
NDP-MSH	[Nle4, dphe7]-α-melanocyte-stimulating hormone
NER	Nucleotide excision repair
PPARγ	Peroxisome proliferator-activated receptor
γH2AX	Phosphorylated histone h2ax
ROS	Reactive oxygen species
SSB	Single-strand break
UVR	Ultraviolet solar radiation
WT	Wildtype
