# Supplementary material for: A Side-by-Side Comparison of Wildtype and Variant Melanocortin 1 Receptor Signaling with Emphasis on Protection against Oxidative Damage to DNA
Source: Int J Mol Sci. 2023 Sep 21;24(18):14381. doi: 10.3390/ijms241814381 (PMC10532403; doi:10.3390/ijms241814381)
Supplement: Supplementary file 1 [file ijms-24-14381-s001.zip › ijms-2551972-supplementary.pdf]

CRISPR-RNA	Target sequence	PAM	Score efficiency	Off-targets
sgRNA1	TCGACACCTCTGGACCGTCC	TGG	92.0	45
sgRNA2	CCAGGACATTTCGACACCTC	TGG	90.7	68
sgRNA3	GAGCTTGGTGGAGAACGCGC	TGG	90.5	79
sgRNA4 (*)	CATCGCCTACTACGACCACG	TGG	98.1	14

Supplementary Table S1. Target and protospacer adjacent motif (PAM) sequences, score efficiency and number of off-targets for each sgRNA. (*) For sgRNA4, primers sequences are: hMC1R_CRISPR_top: 5′-CACCGCATCGCCTACTACGACCACG-3′ and hMC1R_CRISPR_bottom: 5′- AAACCGTGGTCGTAGTAGGCGATGC-3′.

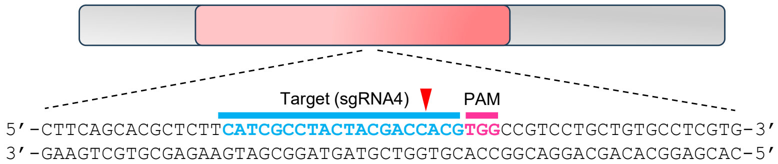

Supplementary Figure 1. Schematic representation of the coding exon (red rectangle: CDS; grey rectangle: full exon) of MC1R gene highlighting the sequence targeted by sgRNA4. This 20 nt sequence pairs with the DNA target (blue bar on top strand), directly upstream of a 5′-NGG adjacent motif (PAM; in pink). Cas9 mediates the DSB ~3 bp upstream of the PAM (red triangle).
